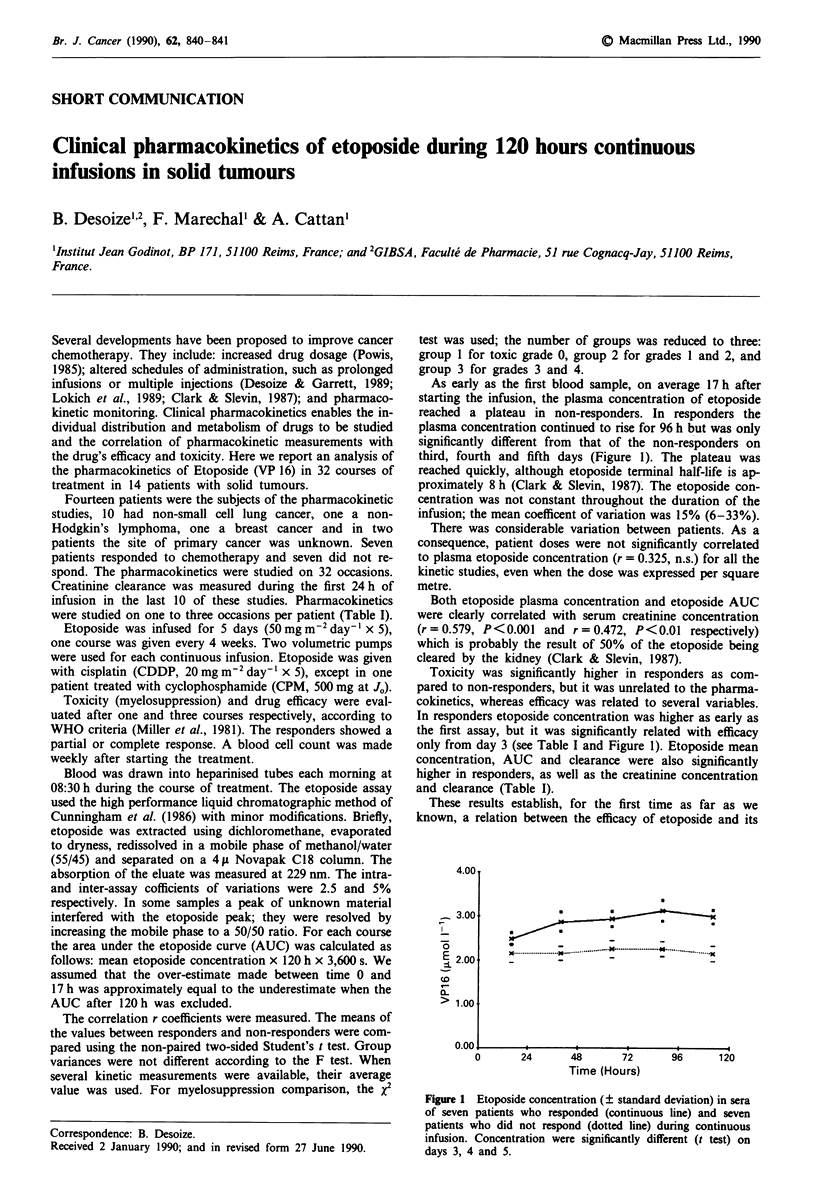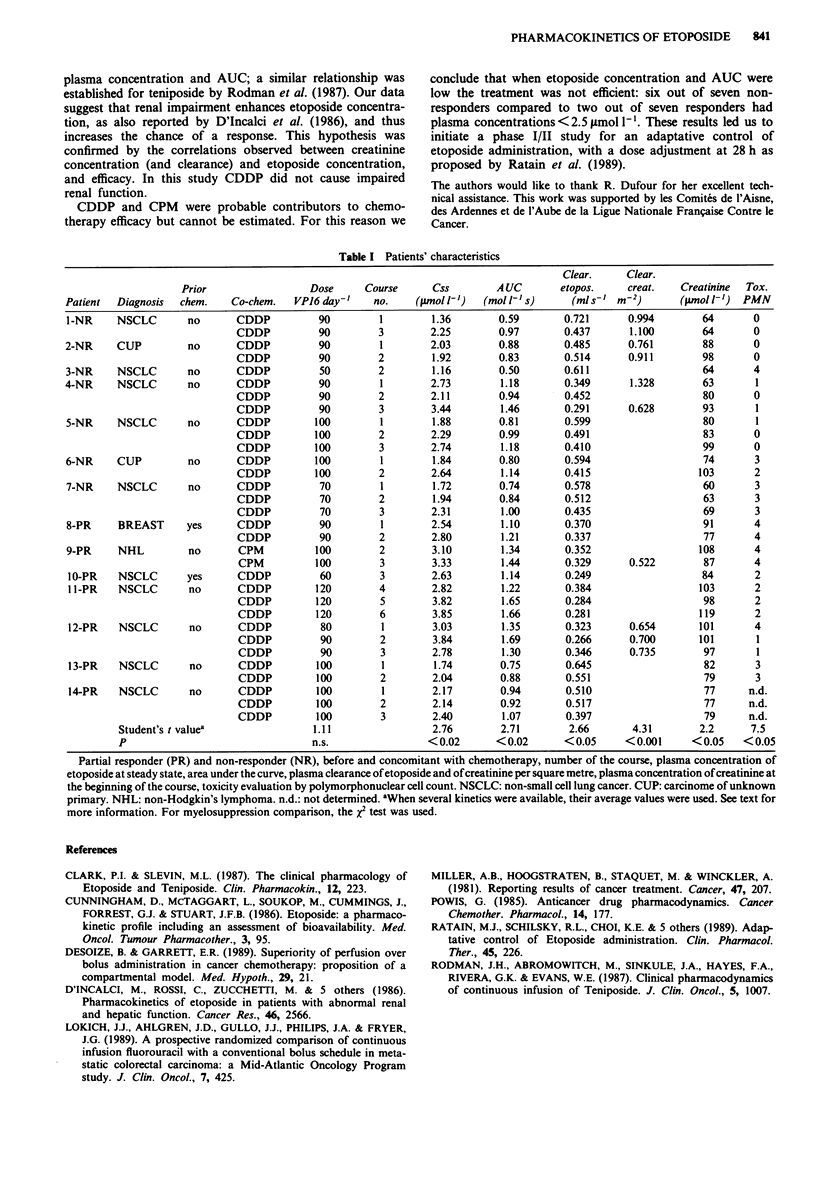# Clinical pharmacokinetics of etoposide during 120 hours continuous infusions in solid tumours.

**DOI:** 10.1038/bjc.1990.390

**Published:** 1990-11

**Authors:** B. Desoize, F. Marechal, A. Cattan

**Affiliations:** Institut Jean Godinot, Reims, France.


Br. J. Cancer (1990), 62, 840-841 © Macmillan Press Ltd., 1990

SHORT COMMUNICATION

### Clinical pharmacokinetics of etoposide during 120 hours continuous infusions in solid tumours

B. Desoize[1,2], F. Marechal[1] & A. Cattan[1]

[1]*Institut Jean Godinot, BP 171, 51100 Reims, France; and* [2]*GIBSA, Faculté de Pharmacie, 51 rue Cognacq-Jay, 51100 Reims, France.*

Several developments have been proposed to improve cancer chemotherapy. They include: increased drug dosage (Powis, 1985); altered schedules of administration, such as prolonged infusions or multiple injections (Desoize & Garrett, 1989; Lokich *et al.*, 1989; Clark & Slevin, 1987); and pharmacokinetic monitoring. Clinical pharmacokinetics enables the individual distribution and metabolism of drugs to be studied and the correlation of pharmacokinetic measurements with the drug's efficacy and toxicity. Here we report an analysis of the pharmacokinetics of Etoposide (VP 16) in 32 courses of treatment in 14 patients with solid tumours.

Fourteen patients were the subjects of the pharmacokinetic studies, 10 had non-small cell lung cancer, one a non-Hodgkin's lymphoma, one a breast cancer and in two patients the site of primary cancer was unknown. Seven patients responded to chemotherapy and seven did not respond. The pharmacokinetics were studied on 32 occasions. Creatinine clearance was measured during the first 24 h of infusion in the last 10 of these studies. Pharmacokinetics were studied on one to three occasions per patient (Table I).

Etoposide was infused for 5 days (50 mg $m^{-2}$ $day^{-1}$ × 5), one course was given every 4 weeks. Two volumetric pumps were used for each continuous infusion. Etoposide was given with cisplatin (CDDP, 20 mg $m^{-2}$ $day^{-1}$ × 5), except in one patient treated with cyclophosphamide (CPM, 500 mg at $J_0$).

Toxicity (myelosuppression) and drug efficacy were evaluated after one and three courses respectively, according to WHO criteria (Miller *et al.*, 1981). The responders showed a partial or complete response. A blood cell count was made weekly after starting the treatment.

Blood was drawn into heparinised tubes each morning at 08:30 h during the course of treatment. The etoposide assay used the high performance liquid chromatographic method of Cunningham *et al.* (1986) with minor modifications. Briefly, etoposide was extracted using dichloromethane, evaporated to dryness, redissolved in a mobile phase of methanol/water (55/45) and separated on a 4 $\mu$ Novapak C18 column. The absorption of the eluate was measured at 229 nm. The intra- and inter-assay cofficients of variations were 2.5 and 5% respectively. In some samples a peak of unknown material interfered with the etoposide peak; they were resolved by increasing the mobile phase to a 50/50 ratio. For each course the area under the etoposide curve (AUC) was calculated as follows: mean etoposide concentration × 120 h × 3,600 s. We assumed that the over-estimate made between time 0 and 17 h was approximately equal to the underestimate when the AUC after 120 h was excluded.

The correlation *r* coefficients were measured. The means of the values between responders and non-responders were compared using the non-paired two-sided Student's *t* test. Group variances were not different according to the F test. When several kinetic measurements were available, their average value was used. For myelosuppression comparison, the $\chi^2$ test was used; the number of groups was reduced to three: group 1 for toxic grade 0, group 2 for grades 1 and 2, and group 3 for grades 3 and 4.

As early as the first blood sample, on average 17 h after starting the infusion, the plasma concentration of etoposide reached a plateau in non-responders. In responders the plasma concentration continued to rise for 96 h but was only significantly different from that of the non-responders on third, fourth and fifth days (Figure 1). The plateau was reached quickly, although etoposide terminal half-life is approximately 8 h (Clark & Slevin, 1987). The etoposide concentration was not constant throughout the duration of the infusion; the mean coefficent of variation was 15% (6–33%).

There was considerable variation between patients. As a consequence, patient doses were not significantly correlated to plasma etoposide concentration ($r = 0.325$, n.s.) for all the kinetic studies, even when the dose was expressed per square metre.

Both etoposide plasma concentration and etoposide AUC were clearly correlated with serum creatinine concentration ($r = 0.579$, $P < 0.001$ and $r = 0.472$, $P < 0.01$ respectively) which is probably the result of 50% of the etoposide being cleared by the kidney (Clark & Slevin, 1987).

Toxicity was significantly higher in responders as compared to non-responders, but it was unrelated to the pharmacokinetics, whereas efficacy was related to several variables. In responders etoposide concentration was higher as early as the first assay, but it was significantly related with efficacy only from day 3 (see Table I and Figure 1). Etoposide mean concentration, AUC and clearance were also significantly higher in responders, as well as the creatinine concentration and clearance (Table I).

These results establish, for the first time as far as we known, a relation between the efficacy of etoposide and its

**Figure 1** Etoposide concentration (± standard deviation) in sera of seven patients who responded (continuous line) and seven patients who did not respond (dotted line) during continuous infusion. Concentration were significantly different (*t* test) on days 3, 4 and 5.

Correspondence: B. Desoize.
Received 2 January 1990; and in revised form 27 June 1990.

plasma concentration and AUC; a similar relationship was established for teniposide by Rodman *et al.* (1987). Our data suggest that renal impairment enhances etoposide concentration, as also reported by D'Incalci *et al.* (1986), and thus increases the chance of a response. This hypothesis was confirmed by the correlations observed between creatinine concentration (and clearance) and etoposide concentration, and efficacy. In this study CDDP did not cause impaired renal function.

CDDP and CPM were probable contributors to chemotherapy efficacy but cannot be estimated. For this reason we conclude that when etoposide concentration and AUC were low the treatment was not efficient: six out of seven non-responders compared to two out of seven responders had plasma concentrations $<2.5\ \mu mol\ l^{-1}$. These results led us to initiate a phase I/II study for an adaptative control of etoposide administration, with a dose adjustment at 28 h as proposed by Ratain *et al.* (1989).

The authors would like to thank R. Dufour for her excellent technical assistance. This work was supported by les Comités de l'Aisne, des Ardennes et de l'Aube de la Ligue Nationale Française Contre le Cancer.

**Table I** Patients' characteristics

| *Patient* | *Diagnosis* | *Prior chem.* | *Co-chem.* | *Dose VP16 day*$^{-1}$ | *Course no.* | *Css* ($\mu mol\ l^{-1}$) | *AUC* ($mol\ l^{-1}\ s$) | *Clear. etopos.* ($ml\ s^{-1}$ | *Clear. creat.* $m^{-2}$) | *Creatinine* ($\mu mol\ l^{-1}$) | *Tox. PMN* |
|---|---|---|---|---|---|---|---|---|---|---|---|
| 1-NR | NSCLC | no | CDDP | 90 | 1 | 1.36 | 0.59 | 0.721 | 0.994 | 64 | 0 |
| | | | CDDP | 90 | 3 | 2.25 | 0.97 | 0.437 | 1.100 | 64 | 0 |
| 2-NR | CUP | no | CDDP | 90 | 1 | 2.03 | 0.88 | 0.485 | 0.761 | 88 | 0 |
| | | | CDDP | 90 | 2 | 1.92 | 0.83 | 0.514 | 0.911 | 98 | 0 |
| 3-NR | NSCLC | no | CDDP | 50 | 2 | 1.16 | 0.50 | 0.611 | | 64 | 4 |
| 4-NR | NSCLC | no | CDDP | 90 | 1 | 2.73 | 1.18 | 0.349 | 1.328 | 63 | 1 |
| | | | CDDP | 90 | 2 | 2.11 | 0.94 | 0.452 | | 80 | 0 |
| | | | CDDP | 90 | 3 | 3.44 | 1.46 | 0.291 | 0.628 | 93 | 1 |
| 5-NR | NSCLC | no | CDDP | 100 | 1 | 1.88 | 0.81 | 0.599 | | 80 | 1 |
| | | | CDDP | 100 | 2 | 2.29 | 0.99 | 0.491 | | 83 | 0 |
| | | | CDDP | 100 | 3 | 2.74 | 1.18 | 0.410 | | 99 | 0 |
| 6-NR | CUP | no | CDDP | 100 | 1 | 1.84 | 0.80 | 0.594 | | 74 | 3 |
| | | | CDDP | 100 | 2 | 2.64 | 1.14 | 0.415 | | 103 | 2 |
| 7-NR | NSCLC | no | CDDP | 70 | 1 | 1.72 | 0.74 | 0.578 | | 60 | 3 |
| | | | CDDP | 70 | 2 | 1.94 | 0.84 | 0.512 | | 63 | 3 |
| | | | CDDP | 70 | 3 | 2.31 | 1.00 | 0.435 | | 69 | 3 |
| 8-PR | BREAST | yes | CDDP | 90 | 1 | 2.54 | 1.10 | 0.370 | | 91 | 4 |
| | | | CDDP | 90 | 2 | 2.80 | 1.21 | 0.337 | | 77 | 4 |
| 9-PR | NHL | no | CPM | 100 | 2 | 3.10 | 1.34 | 0.352 | | 108 | 4 |
| | | | CPM | 100 | 3 | 3.33 | 1.44 | 0.329 | 0.522 | 87 | 4 |
| 10-PR | NSCLC | yes | CDDP | 60 | 3 | 2.63 | 1.14 | 0.249 | | 84 | 2 |
| 11-PR | NSCLC | no | CDDP | 120 | 4 | 2.82 | 1.22 | 0.384 | | 103 | 2 |
| | | | CDDP | 120 | 5 | 3.82 | 1.65 | 0.284 | | 98 | 2 |
| | | | CDDP | 120 | 6 | 3.85 | 1.66 | 0.281 | | 119 | 2 |
| 12-PR | NSCLC | no | CDDP | 80 | 1 | 3.03 | 1.35 | 0.323 | 0.654 | 101 | 4 |
| | | | CDDP | 90 | 2 | 3.84 | 1.69 | 0.266 | 0.700 | 101 | 1 |
| | | | CDDP | 90 | 3 | 2.78 | 1.30 | 0.346 | 0.735 | 97 | 1 |
| 13-PR | NSCLC | no | CDDP | 100 | 1 | 1.74 | 0.75 | 0.645 | | 82 | 3 |
| | | | CDDP | 100 | 2 | 2.04 | 0.88 | 0.551 | | 79 | 3 |
| 14-PR | NSCLC | no | CDDP | 100 | 1 | 2.17 | 0.94 | 0.510 | | 77 | n.d. |
| | | | CDDP | 100 | 2 | 2.14 | 0.92 | 0.517 | | 77 | n.d. |
| | | | CDDP | 100 | 3 | 2.40 | 1.07 | 0.397 | | 79 | n.d. |
| | Student's *t* value[a] | | | 1.11 | | 2.76 | 2.71 | 2.66 | 4.31 | 2.2 | 7.5 |
| | *P* | | | n.s. | | $<0.02$ | $<0.02$ | $<0.05$ | $<0.001$ | $<0.05$ | $<0.05$ |

Partial responder (PR) and non-responder (NR), before and concomitant with chemotherapy, number of the course, plasma concentration of etoposide at steady state, area under the curve, plasma clearance of etoposide and of creatinine per square metre, plasma concentration of creatinine at the beginning of the course, toxicity evaluation by polymorphonuclear cell count. NSCLC: non-small cell lung cancer. CUP: carcinome of unknown primary. NHL: non-Hodgkin's lymphoma. n.d.: not determined. [a]When several kinetics were available, their average values were used. See text for more information. For myelosuppression comparison, the $\chi^2$ test was used.

### References

CLARK, P.I. & SLEVIN, M.L. (1987). The clinical pharmacology of Etoposide and Teniposide. *Clin. Pharmacokin.*, **12**, 223.

CUNNINGHAM, D., MCTAGGART, L., SOUKOP, M., CUMMINGS, J., FORREST, G.J. & STUART, J.F.B. (1986). Etoposide: a pharmacokinetic profile including an assessment of bioavailability. *Med. Oncol. Tumour Pharmacother.*, **3**, 95.

DESOIZE, B. & GARRETT, E.R. (1989). Superiority of perfusion over bolus administration in cancer chemotherapy: proposition of a compartmental model. *Med. Hypoth.*, **29**, 21.

D'INCALCI, M., ROSSI, C., ZUCCHETTI, M. & 5 others (1986). Pharmacokinetics of etoposide in patients with abnormal renal and hepatic function. *Cancer Res.*, **46**, 2566.

LOKICH, J.J., AHLGREN, J.D., GULLO, J.J., PHILIPS, J.A. & FRYER, J.G. (1989). A prospective randomized comparison of continuous infusion fluorouracil with a conventional bolus schedule in metastatic colorectal carcinoma: a Mid-Atlantic Oncology Program study. *J. Clin. Oncol.*, **7**, 425.

MILLER, A.B., HOOGSTRATEN, B., STAQUET, M. & WINCKLER, A. (1981). Reporting results of cancer treatment. *Cancer*, **47**, 207.

POWIS, G. (1985). Anticancer drug pharmacodynamics. *Cancer Chemother. Pharmacol.*, **14**, 177.

RATAIN, M.J., SCHILSKY, R.L., CHOI, K.E. & 5 others (1989). Adaptative control of Etoposide administration. *Clin. Pharmacol. Ther.*, **45**, 226.

RODMAN, J.H., ABROMOWITCH, M., SINKULE, J.A., HAYES, F.A., RIVERA, G.K. & EVANS, W.E. (1987). Clinical pharmacodynamics of continuous infusion of Teniposide. *J. Clin. Oncol.*, **5**, 1007.